# Exploration of carbohydrate binding behavior and anti-proliferative activities of *Arisaema tortuosum* lectin

**DOI:** 10.1186/s12867-019-0132-0

**Published:** 2019-05-07

**Authors:** Kshema Thakur, Tarnjeet Kaur, Manpreet Kaur, Rachna Hora, Jatinder Singh

**Affiliations:** 1 Department of Molecular Biology & Biochemistry, Guru Nanak Dev University, Amritsar, Punjab 143005 India; 2 Department of Human Genetics, Guru Nanak Dev University, Amritsar, Punjab 143005 India; 3 Present Address: Department of Biochemistry, Dr. Y.S.P. Govt. Medical College, Distt. Sirmaur, Nahan, H.P 173001 India

**Keywords:** Apoptosis, Complex *N*-glycans, Glycan array, ITC, Lectin, Molecular cloning

## Abstract

**Background:**

Lectins have come a long way from being identified as proteins that agglutinate cells to promising therapeutic agents in modern medicine. Through their specific binding property, they have proven to be anti-cancer, anti-insect, anti-viral agents without affecting the non-target cells. The *Arisaema tortuosum* lectin (ATL) is a known anti-insect and anti-cancer candidate, also has interesting physical properties. In the present work, its carbohydrate binding behavior is investigated in detail, along with its anti-proliferative property.

**Results:**

The microcalorimetry of ATL with a complex glycoprotein asialofetuin demonstrated trivalency contributed by multiple binding sites and enthalpically driven spontaneous association. The complex sugar specificity of ATL towards multiple sugars was also demonstrated in glycan array analysis in which the trimannosyl pentasaccharide core *N*-glycan [Manα1-6(Manα1-3)Manβ1-4GlcNAcβ1-4GlcNAcβ] was the highest binding motif. The high binding glycans for ATL were high mannans, complex *N*-glycans, core fucosylated *N*-glycans and glycans with terminal lactosamine units attached to pentasaccharide core. ATL induced cell death in IMR-32 cells was observed as time dependent loss in cell number, formation of apoptotic bodies and DNA damage. As a first report of molecular cloning of ATL, the in silico analysis of its cDNA revealed ATL to be a *β*-sheet rich heterotetramer. A homology model of ATL showed beta prism architecture in each monomer with 85% residues in favoured region of Ramachandran plot.

**Conclusions:**

Detailed exploration of carbohydrate binding behavior indicated ATL specificity towards complex glycans, while no binding to simple sugars, including mannose. Sequence analysis of ATL cDNA revealed that during the tandem evolutionary events, domain duplication and mutations lead to the loss of mannose specificity, acquiring of new sugar specificity towards complex sugars. It also resulted in the formation of a two-domain single chain polypeptide with both domains having different binding sites due to mutations within the consensus carbohydrate recognition sites [QXDXNXVXY]. This unique sugar specificity can account for its significant biological properties. Overall finding of present work signifies anti-cancer, anti-insect and anti-viral potential of ATL making it an interesting molecule for future research and/or theragnostic applications.

**Electronic supplementary material:**

The online version of this article (10.1186/s12867-019-0132-0) contains supplementary material, which is available to authorized users.

## Background

Enormous amount of literature affirmatively suggests that aberrant glycosylation is a classic hallmark of the malignant transformation and the full clinical potential of this hallmark in cancer diagnosis and therapeutics needs to be efficiently realized towards meeting better treatment goals [1–4]. Plant lectins are amongst the most extensively studied natural molecules which have anti-cancer potential [1, 5–7]. Pre-clinical trials with ConA and *Momordica charantia* lectin [6, 8] and clinical trials with Mistletoe lectins (extensively reviewed by Marvibaigi et al. [9]) have been conducted successfully. Although, with a size large enough to be capable of generating immunogenic response, they are instead, becoming favourites in alternative therapies. This is due to their highly specific binding nature which remains central to their various activities. Owing to this, lectins are known to bind directly to specific cells [10].

Based on available plant genome/transcriptome sequences, common carbohydrate recognition domains (CRD) were identified across taxonomical families defining modern lectinology [11]. Having common sequence and structural features, lectins were classified into 12 families each having a characteristic CRD. Each CRD has a characteristic structure formed by a typical lectin fold and conserved amino acid sequence. One such family based on CRD is *Galanthus nivalis* agglutinin (GNA) related lectins. These are interesting lectins that exhibit diverse biological potentials including anti-tumor activity, anti-insect potential and anti-viral properties [7, 12–15]. An important taxonomical family of this group is Araceae. A rich family of pan-tropical herbs and vines with underground tubers or rhizomes, Araceae is reported to comprise of no less than 125 genera and 3750 species [16] showing diverse properties such as anti-viral, anti-proliferative, anti-insect and anti-microbial properties [7, 12–15]. Despite the structural homology and similar sugar specificity, each Araceae lectin is unique in its activity and potential which needs to be targeted individually to realize their full potential.

The present work is a first report of molecular cloning of an anti-cancer lectin *Arisaema tortuosum* (ATL) belonging to GNA-related lectin superfamily. ATL, a 52 kDa tetrameric protein was reported to show anti-proliferative and anti-insect potential in its first report from our laboratory [17]. Physical characterization of the lectin revealed that it is thermally stable, resistant to acid, alkali and denaturant treatment and demonstrated a molten globule structure [18]. Immediate work presents extensive studies of lectin–glycan interaction of ATL. Isothermal titration microcalorimetry (ITC) was employed to evaluate the binding thermodynamics with a glycoconjugate. ATL binding to over 600 glycans was characterized in a microarray. In order to investigate the molecular basis of the binding pattern of ATL with its ligands, molecular cloning was performed to analyse its sequence. Present work is the first report of detailed investigation of lectin–carbohydrate interaction of ATL.

## Results

### Lectin–carbohydrate interaction

#### Isothermal titration microcalorimetry

**Fig. 1:**
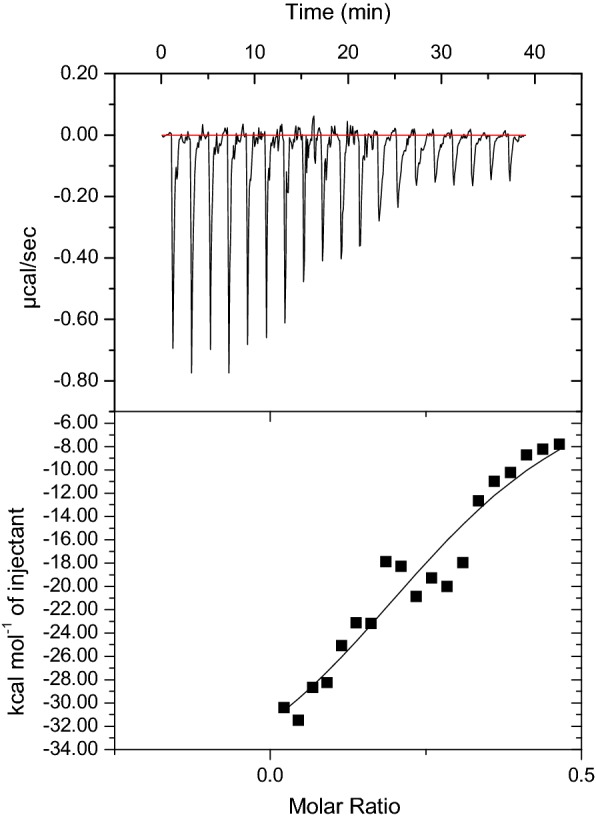
Enthalpogram of Afet interaction with ATL. Upper panel shows raw ITC data obtained after each automatic injection (18 injections). Lower panel displays integrated curve comprising experimental data points (filled squares) and best fit (curved line) with one site model

Various thermodynamic parameters characterizing ligand (Asialofetuin, Afet) binding of ATL are provided in Additional file 1: Table S1. Binding pattern of Afet with ATL demonstrates cooperativity as is visible from a sigmoidal curve (Fig. 1). It can be clearly seen that through successive injections, saturation is achieved and a pattern of monotonic decrease in the exothermic heat of ATL–Afet binding is observed. ATL–Afet binding demonstrates an exothermic reaction in which the heat released is a function of ATL–Afet molar ratio.

#### Glycan array analysis

**Fig. 2:**
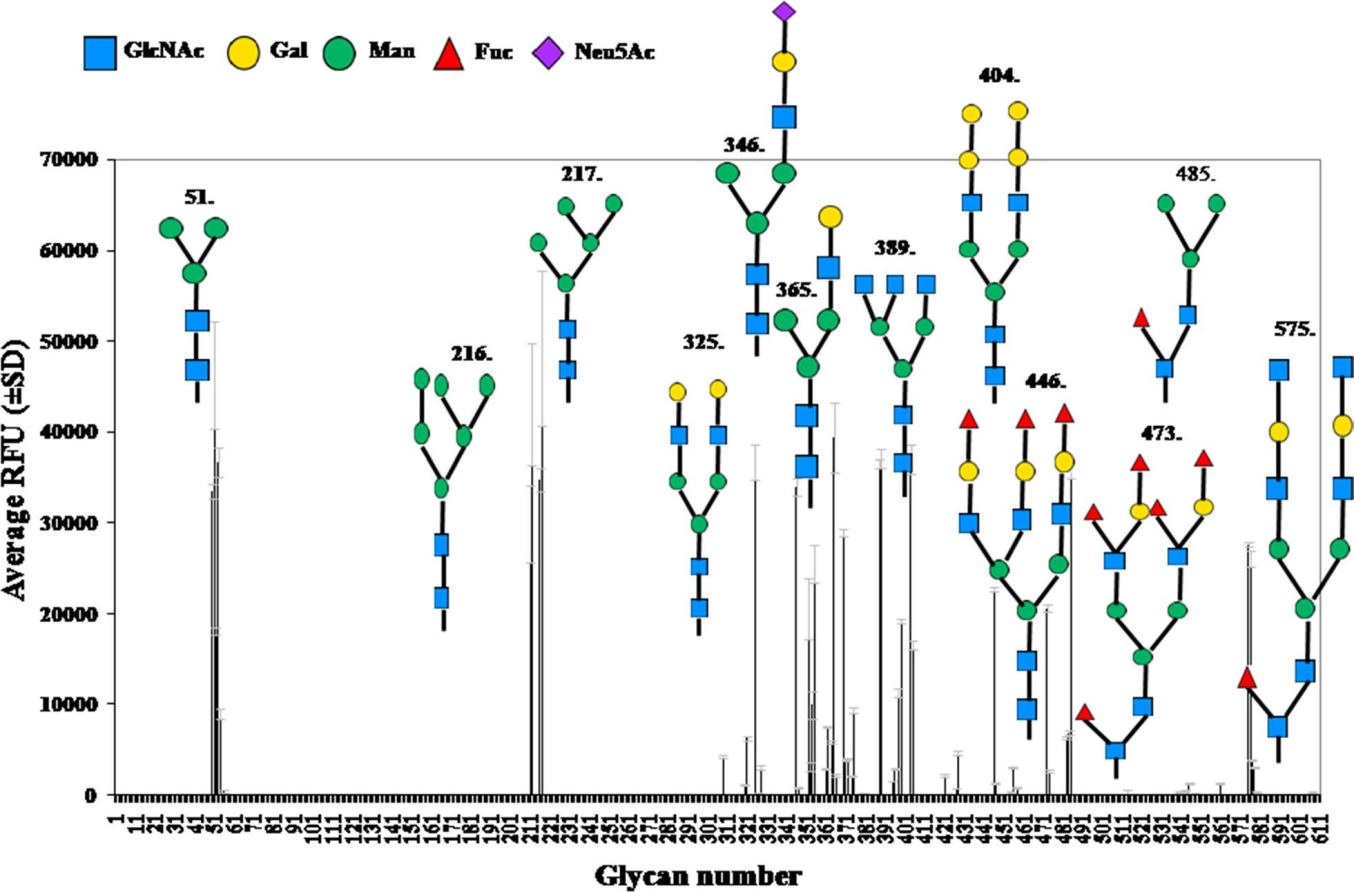
Glycan array analysis of biotinylated ATL. Graph presented here is for 10 µg ml^−1^ lectin concentration. Binding intensity of lectin to glycans was expressed as RFU. Error bars represent mean ± standard deviation. Structures of strongest binding glycans according to ranking analysis and their respective numbers in the array are given. The symbols used for constructing structures are from Essentials of Glycobiology, 3rd edition, Appendix 1B: “Essentials Symbol Nomenclature” for Graphical Representations of Glycans (PMID 26543186)

The output for glycan array analysis was graphically represented for each glycan as average relative fluorescence unit (RFU) with standard deviation (Fig. 2). Some of the glycans having highest average RFUs are provided in representative structures in the same graph. In this graph, fluorescence intensities of ATL provided are at 10 µg ml^−1^ concentration. The primary data for other concentrations of ATL are available at www.functionalglycomics.org. Both glycopattern and ranking analysis helped identify around 27 glycans which had > 10 average rank (Table 1). Glycopattern helped segregate all the glycans tested at various concentrations into motifs, binders and non-binders for ATL (Table 2). The most common motif for ATL was glycan no. 51sp13 [Manα1-6(Manα1-3)Manβ1-4GlcNAcβ1-4GlcNAcβ], the core pentasaccharide with 88% percentile rank. Other strong binders for ATL also belonged to the group of core *N*-glycans. It was observed that binding was affected with addition of groups to the core pentasccharide forming subcategories of highest binders as high mannose, complex and fucosylated core *N*-glycans.

**Table 1:** Binding specificity of ATL towards various glycans as determined by microarray

Glycan no.	Glycan structure	Avg. rank^a^
High mannose *N*-glycans		
51	Mana1-6(Mana1-3)Manb1-4GlcNAcb1-4GlcNAcb-Sp13	87.6
217	Mana1-6(Mana1-3)Mana1-6(Mana1-3)Manb1-4GlcNAcb1-4GlcNAcb-Sp12	67.9
50	Mana1-6(Mana1-3)Manb1-4GlcNAcb1-4GlcNAcb-Sp12	67.1
485	Mana1-6(Mana1-3)Manb1-4GlcNAcb1-4(Fuca1-6)GlcNAcb-Sp19	62.5
216	Mana1-6(Mana1-3)Mana1-6(Mana1-2Mana1-3)Manb1-4GlcNAcb1-4GlcNAcb-Sp12	37.3
212	Mana1-2Mana1-6(Mana1-3)Mana1-6(Mana1-2Mana1-2Mana1-3)Manb1-4GlcNAcb1-4GlcNAcb-Sp12	35.7
211	Mana1-6(Mana1-2Mana1-3)Mana1-6(Mana1-2Mana1-3)Manb1-4GlcNAcb1-4GlcNAcb-Sp12	30.4
Complex *N*-glycans		
389	GlcNAcb1-2Mana1-6(GlcNAcb1-4(GlcNAcb1-2)Mana1-3)Manb1-4GlcNAcb1-4GlcNAc-Sp21	85.5
404	Gala1-4Galb1-3GlcNAcb1-2Mana1-6(Gala1-4Galb1-3GlcNAcb1-2Mana1-3)Manb1-4GlcNAcb1-4GlcNAcb-Sp19	70.8
365	Galb1-4GlcNAcb1-2Mana1-6(Mana1-3)Manb1-4GlcNAcb1-4GlcNAcb-Sp12	59.9
325	Galb1-3GlcNAcb1-2Mana1-6(Galb1-3GlcNAcb1-2Mana1-3)Manb1-4GlcNAcb1-4GlcNAcb-Sp19	57.7
388	Galb1-4GlcNAcb1-6(Galb1-4GlcNAcb1-2)Mana1-6(Galb1-4GlcNAcb1-4(Galb1-4GlcNAcb1-2)Mana1-3)Manb1-4GlcNAcb1-4GlcNAcb-Sp21	51.9
53	GlcNAcb1-2Mana1-6(GlcNAcb1-2Mana1-3)Manb1-4GlcNAcb1-4GlcNAcb-Sp13	48.0
446	Fuca1-2Galb1-4 GlcNAcb1-2Mana1-6(Fuca1-2Galb1-4GlcNAcb1-2(Fuca1-2Galb1-4GlcNAcb1-4)Mana1-3)Manb1-4GlcNAcb1-4GlcNAcb-Sp12	25.9
370	Galb1-4GlcNAcb1-2Mana1-6(Galb1-4GlcNAcb1-4(Galb1-4GlcNAcb1-2)Mana1-3)Manb1-4GlcNAcb1-4GlcNAc-Sp21	24.5
352	Mana1-6(Galb1-4GlcNAcb1-2Mana1-3)Manb1-4GlcNAcb1-4GlcNAcb-Sp12	15.5
52	GlcNAcb1-2Mana1-6(GlcNAcb1-2Mana1-3)Manb1-4GlcNAcb1-4GlcNAcb-Sp12	14.9
399	Galb1-4GlcNAcb1-2Mana1-6(GlcNAcb1-2Mana1-3)Manb1-4GlcNAcb1-4GlcNAc-Sp12	13.8
405	Gala1-4Galb1-4GlcNAcb1-2Mana1-6(Gala1-4Galb1-4GlcNAcb1-2Mana1-3)Manb1-4GlcNAcb1-4GlcNAcb-Sp24	11.8
Fucosylated core *N*-glycans		
355	Galb1-3GlcNAcb1-2Mana1-6(Galb1-3GlcNAcb1-2Mana1-3)Manb1-4GlcNAcb1-4(Fuca1-6)GlcNAcb-Sp22	24.1
576	Galb1-4GlcNAcb1-3Galb1-4GlcNAcb1-2Mana1-6(Galb1-4GlcNAcb1-3Galb1-4GlcNAcb1-2Mana1-3)Manb1-4GlcNAcb1-4(Fuca1-6)GlcNAcb-Sp24	20.9
575	GlcNAcb1-3Galb1-4GlcNAcb1-2Mana1-6(GlcNAcb1-3Galb1-4GlcNAcb1-2Mana1-3)Manb1-4GlcNAcb1-4(Fuca1-6)GlcNAcb-Sp24	20.6
473	Fuca1-2Galb1-4(Fuca1-3)GlcNAcb1-2Mana1-6(Fuca1-2Galb1-4(Fuca1-3)GlcNAcb1-2Mana1-3)Manb1-4GlcNAcb1-4(Fuca1-6)GlcNAcb-Sp24	20.0

^a^Average rank is based on analysis of RFU for three concentrations of lectin as discussed in text

**Table 2:** Glycopattern findings for ATL

Experiment (v5.0)	Motifs	Binders	Non binders
ATL	3	25	586
ATL (10.0 µg ml^−1^)^a^	3	28	583
ATL (1.0 µg ml^−1^)^a^	3	14	597
ATL (0.1 µg ml^−1^)^a^	3	15	596

^a^Indicates the ATL concentration in specific experiment

#### Molecular cloning

##### Full length cloning and bioinformatic characterization

Rapid amplification of cDNA ends polymerase chain reaction (RACE-PCR) was used to synthesize the full length cDNA of ATL total RNA isolated from tubers. The 3′ and 5′ RACE products were 595 bp and 662 bp respectively making a final full length clone of 1099 bp with a coding sequence of 774 bp (accession no. KX132810) (see Additional file 2: Figure S1). The full length clone had a 60% of GC component with 3 novel polyadenylation signal sequences in 3′UTR. BLASTn showed 80% homology of ATL cDNA with various Araceae members. The ATL precursor protein was deduced to be of 257 residues (GenBank accession no.: APQ47296) (see Additional file 2: Figure S1) with a predicted molecular weight of 28.1 kDa. The precursor protein was presumed to have a 23 residue signal peptide with a cleavage site between residue 23 and 24 (AAA-VG). Physicochemical characters of this predicted protein are summarized in Additional file 3: Table S2. The deduced amino acid sequence of ATL showed 91–51% homology to other Alismatales lectins whereas only 38–30% homology to non-Alismatales members of GNA-related lectin superfamily.

**Fig. 3:**
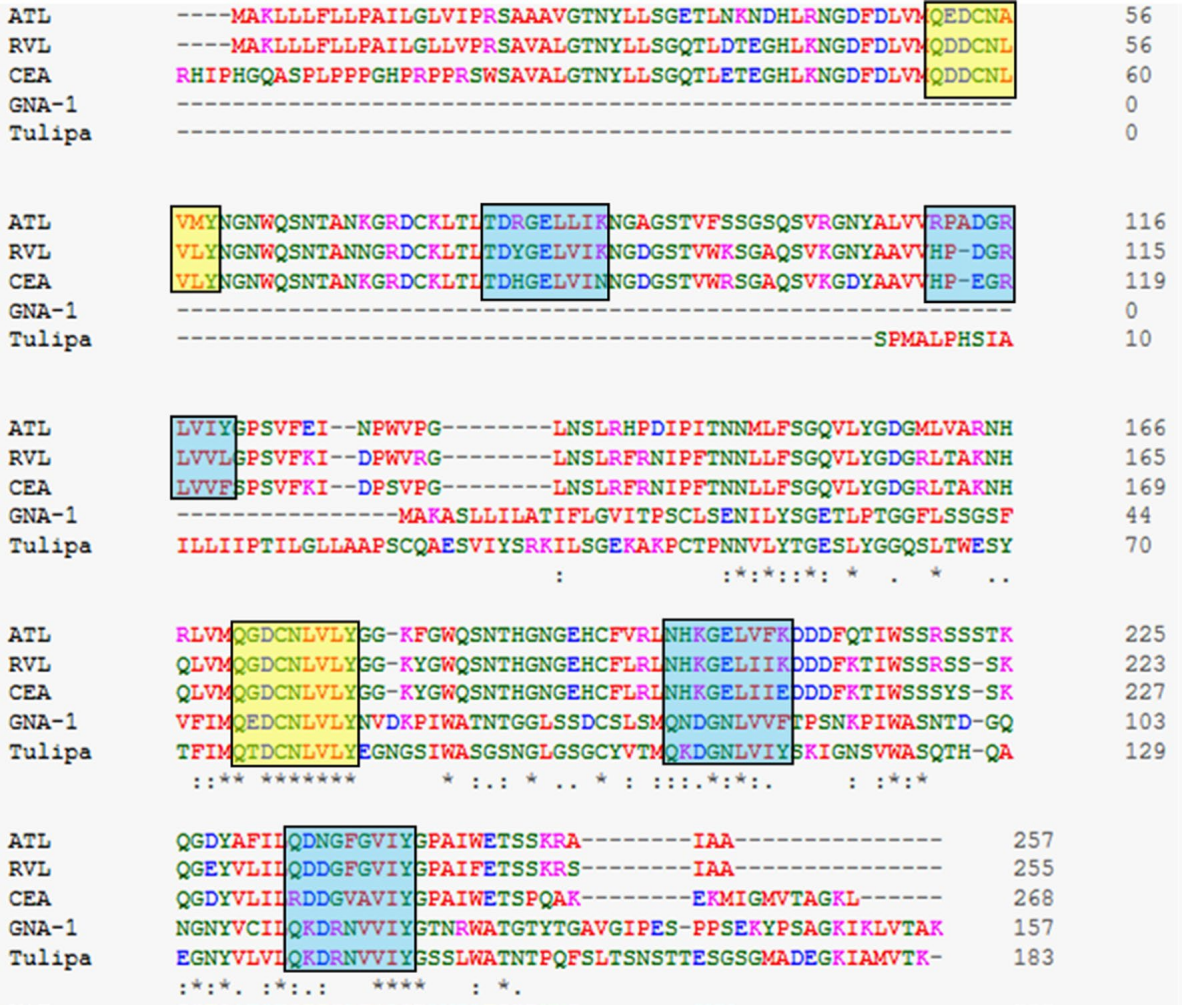
Multiple sequence alignment showing conserved and lost CRS. Yellow boxed regions show conserved CRS while blue boxed regions show lost CRS in Araceae members in comparison to other mannose binding lectins

**Fig. 4:**
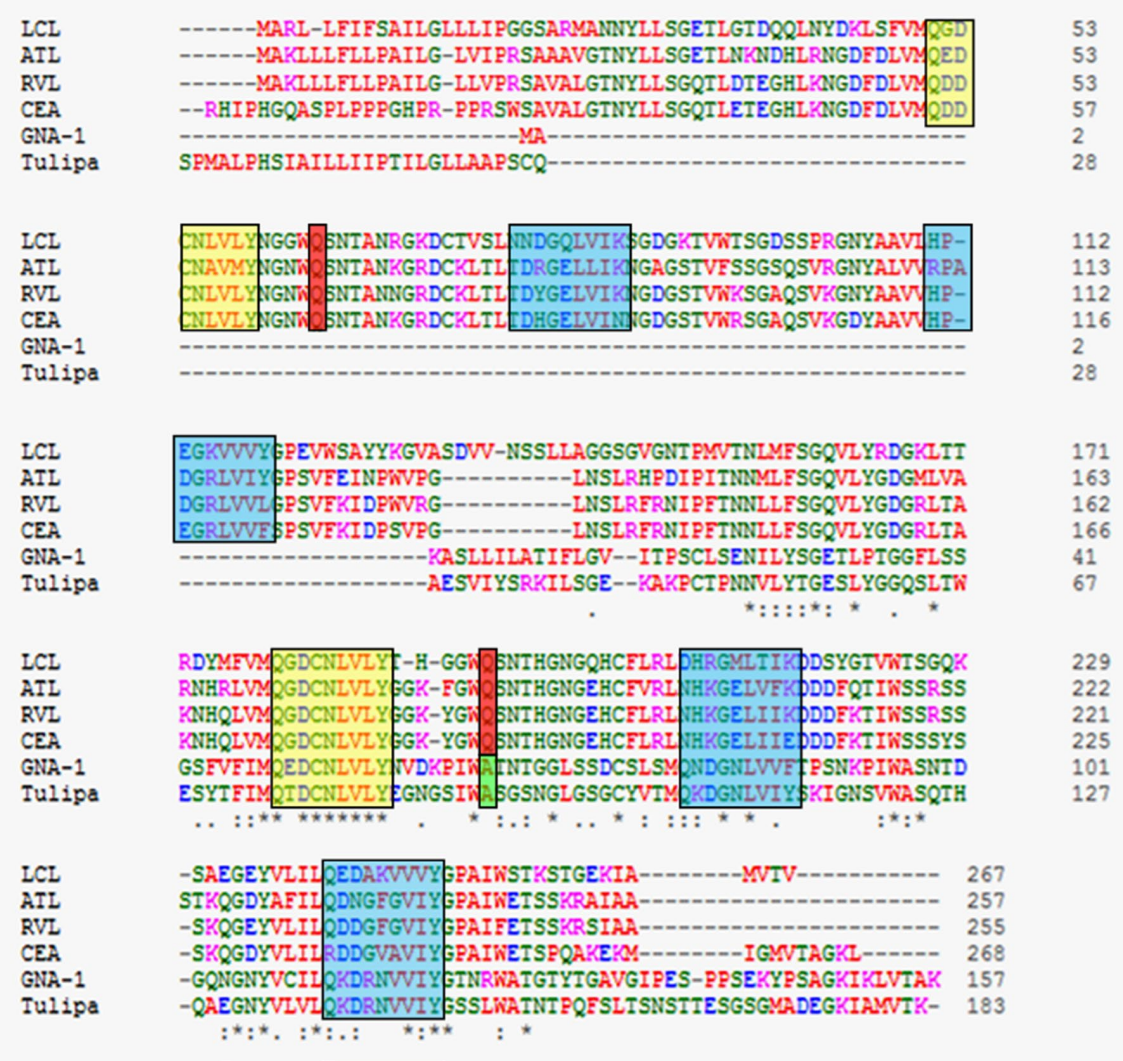
Sequence alignment of ATL with mannose binding (GNA, Tulipa) and non-mannose binding (Araceae) lectins from GNA-related lectin superfamily. Conserved CRS are highlighted in yellow boxes. Blue boxes highlight the CRS mutated in non-mannose binding Araceae lectins. Substitution of a conserved residue downstream of conserved CRS is highlighted in red box as against conserved residue in green. Accession numbers of lectins and their full forms are provided in Additional file 4: Table S3

Both Prosite and CDD aided in understanding the function of ATL on the basis of its sequence based on available data and classified ATL as Bulb_lectin (Prosite ID: PS50927) and Bulb-type mannose-specific lectin (CDD accession no. cd00028) with two such domains in the polypeptide. In the two predicted domains, N_26_–P_132_ constituted DOM1 (106 amino acids) and N_146_–A_254_ constituted DOM2 (108 amino acids) (see Additional file 2: Figure S1). The subunit molecular mass of DOM1 and DOM2 was 11.6 and 12.1 kDa respectively. The DOM1 and DOM2 did not share any significant similarity amongst the two. The bulb_lectin domain has a well defined beta-prism architecture giving the lectins of this group name BP2 lectins. The BP2 lectins share a common feature of having three-fold internal repeats three in number with a consensus sequence of [QXDXNXVXY] constituting three independent mannose binding motifs as with GNA, the representative lectin of this group. This sequence has an extended consensus of [ZQXDXNZVZY] where Z is a hydrophobic amino acid and N-terminal Z is specifically any residue amongst M/I/L/V [19]. These authors further asserted that the hydrophobic triplet near the 3′ of the consensus plays a critical role in mannose binding, facilitating strong van der Waals interactions with α-d-mannose at C4 and C6. The ATL sequence demonstrates one such consensus sequence conserved in each domain. It also has the extended consensus in carbohydrate recognition site (CRS) and the hydrophobic triplet (Fig. 3). On alignment of ATL sequence with some other BP2 lectins however, highlighted certain variations in the CRS of ATL and also that in contrast to three conserved CRS in other lectins, ATL has only one per domain (Fig. 4).

##### Structural prediction

A β-sheet rich structure was predicted for ATL with a *β* component of 51.36% and α-helical component of only 8.95% with the rest 39.69% as random coil. A total of 138 templates were returned for ATL by SWISS-MODEL search. Of all the submitted structures, ATL showed highest homology to lectins *Remusatia vivipara* (RVL, PDB ID: 3R0E) and *Colocasia esculenta* (CEA). Both submitted structures of CEA, one with ions (PDB ID: 5D5G) and another with mannose (PDB ID: 5D9Z) were used as templates for ruling out any variation in structure due to presence of complexed molecules. The ATL homology model with 3R0E as template was considered to be the best based on the various validation indices. Additional file 5: Figure S2A shows the cartoon representation of predicted theoretical model of ATL. The predicted model of ATL had a good ERRAT value (69.03%), VERIFY3D (97.44%) and Z-score (− 6.47) and predominantly negative energy plots (see Additional file 5: Figure S2B, C). According to PROCHECK, as much as 85.4% of predicted ATL residues were in the most favoured region of Ramachandran Plot (see Additional file 5: Figure S2D). There were two residues Asn37 and Ser228 of the model that were predicted in the generously disallowed region of the plot and were located in the loops. The G-factor contributed by dihedral angles and main-chain covalent forces were − 0.17 and − 0.42 respectively.

##### Phylogenetic analysis

**Fig. 5:**
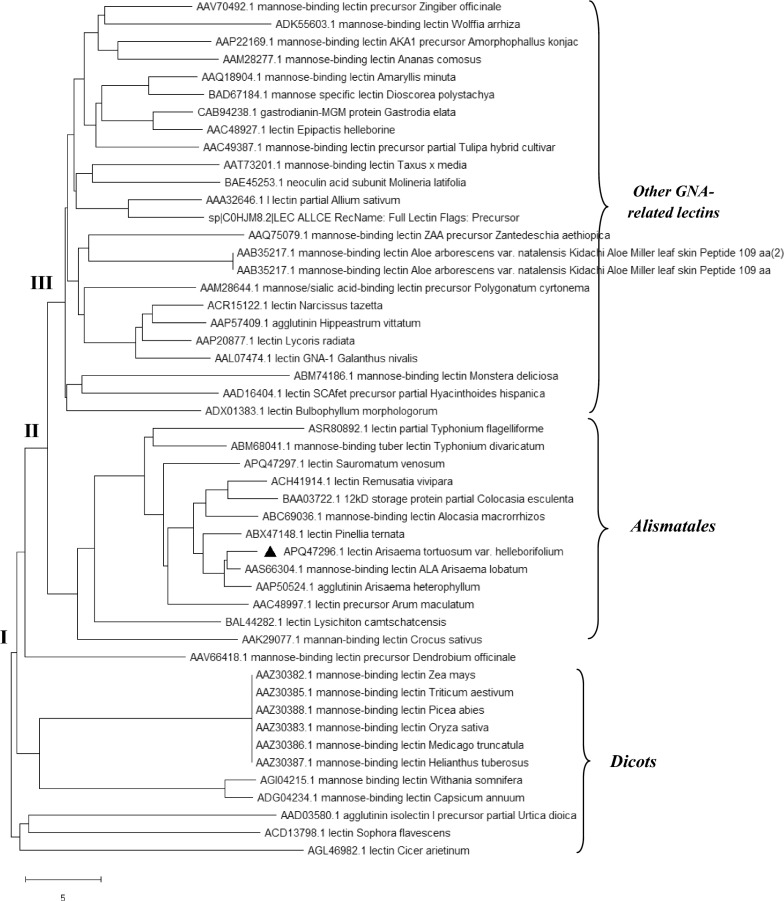
Evolutionary relationship of ATL (highlighted with filled diamond label) with other phytolectins having CRS as inferred using the Neighbor-Joining method. The tree is drawn to scale, with branch lengths in the same units as those of the evolutionary distances used to infer the phylogenetic tree. All positions containing gaps and missing data were eliminated

The GNA related lectins included in the present study (49 taxa) showed obvious taxonomical divisions (Fig. 5) with two major branches of dicots and monocots (node I). The Dicot members were Solanales, Asterales, Fabales and Rosales. The only non-dicots in this group were lectins from Poales and Pinales. Amongst the monocots, the lectins segregated into two major branches at node II in Alismatales lectins and all the other monocot lectins included in the study. The lectins from order Alismatales belonged to family Araceae specifically. ATL belonged to this Alismatales branch. The only non-Alismatales lectin clustering in this branch was *Crocus sativus* of order Asparagales. Lectins belonging to all the other orders Asparagales, Zingiberales, Liliales, Poales and Dioscorales (here marked under ‘other GNA related lectins’) and one gymnospermous order Pinales clustered in the branch III. A few lectins from Alismatales and a dicot also clustered within this branch.

### Anti-proliferative activity

#### MTT assay

There was no inhibitory effect on cell proliferation on the treatment of A498 (kidney carcinoma) cells. ATL showed 24.38% proliferation inhibition towards A549 whereas it was non-responsive towards HeLa cell line. It showed 85% inhibitory potential towards IMR-32 neuroblastoma cell line.

#### Trypan blue assay

**Fig. 6:**
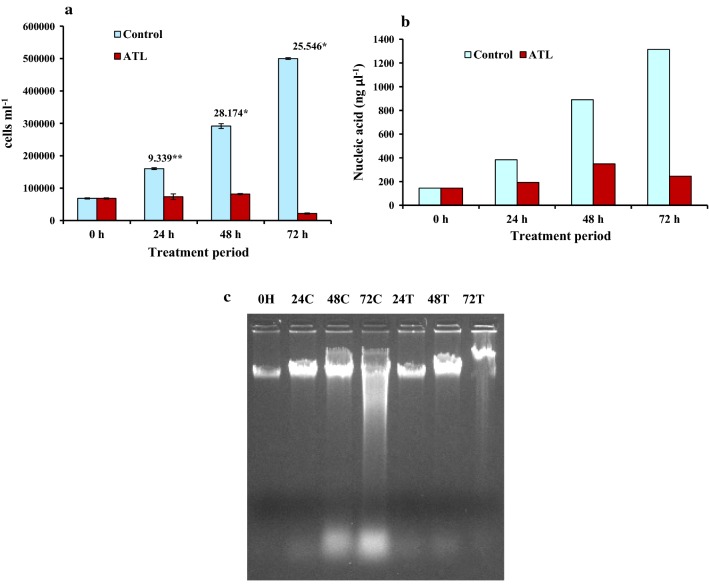
Effect on IMR-32 cells of ATL treatment on: **a** the cell number as studied on time dependent lectin treatment in trypan blue dye exclusion assay. Values above the bars are *t*-values of comparison of cell number between control and treatment. Error bars indicate standard error. Significance levels are provided in the text. **b** Nucleic acid content of the cells. Best results out of the three experiments performed are presented. **c** Agarose gel pictures showing DNA damage. Labels above the lanes represent respective control and treatment stages

Treatment of IMR 32 cells had a negative effect on cell viability reducing the cell number as compared to control cells. The difference in the cell number of control and treatment was statistically significant with *t*-values of 9.339*,^1^ 28.174**^2^ and 25.546** for 24 h, 48 h and 72 h of treatment (Fig. 6a).

#### Nucleic acid content analysis and DNA damage assay

Total nucleic acid content of cells was also affected in a time dependent treatment (Fig. 6b). At the start of experiment, total nucleic acid content was 4.36 μg. After, 24 h of treatment, it was 5.77 μg against 11.53 μg of control cells. After 48 h of treatment, while total DNA isolated from control cells showed an appreciable increase to 26.71 μg, it was only 10.46 μg for treated cells. While, DNA isolated from control cells after 72 h of treatment was further increased to 39.41 μg, considerable effect was observed in case of ATL treated cells, with total DNA content decreasing to 7.37 μg only. A loss in the DNA content could clearly be observed in case of ATL treated cells in agarose gels (Fig. 6c).

#### Morphology assay

**Fig. 7:**
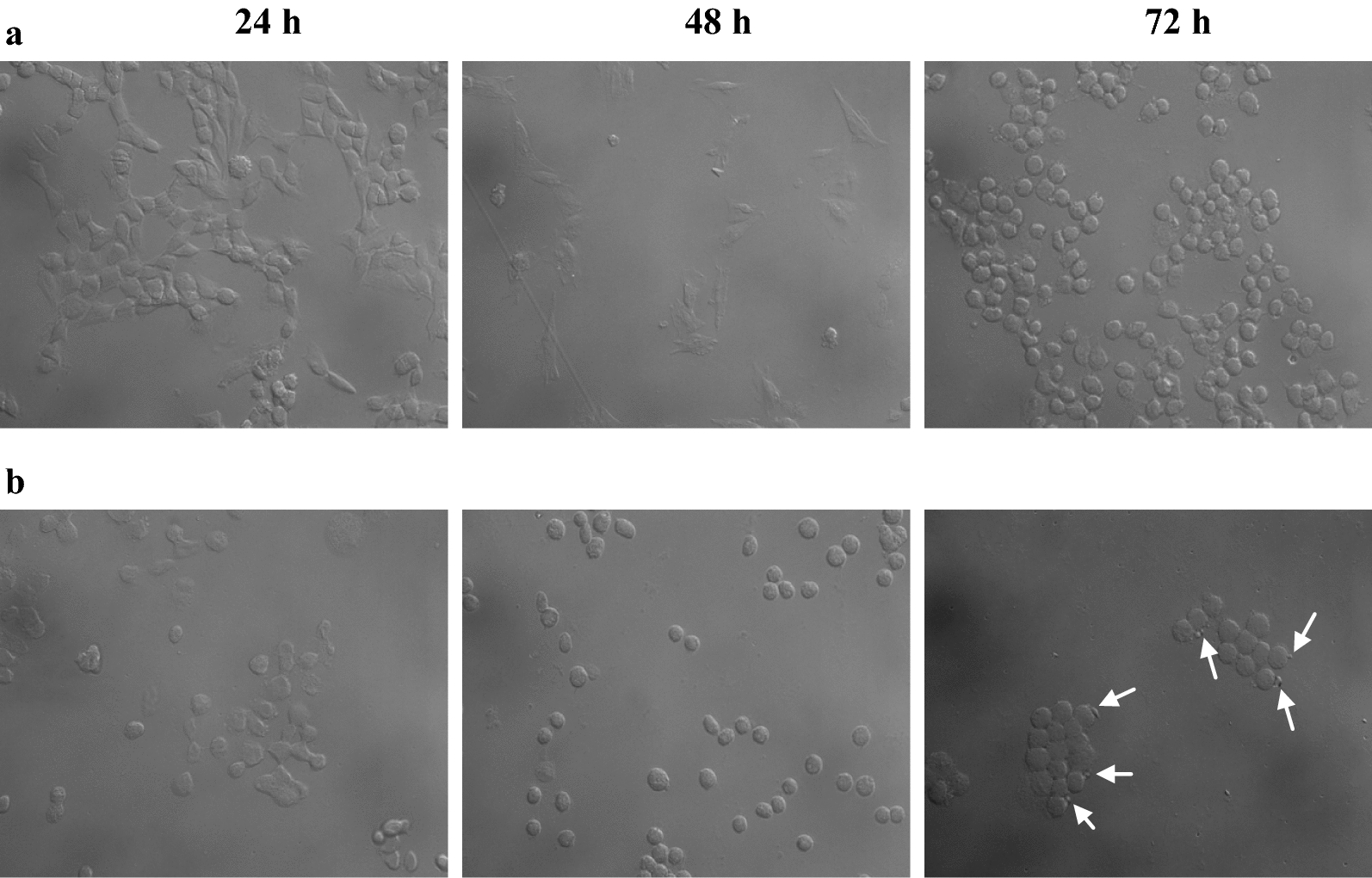
Morphology of IMR-32 cells as observed under phase contrast microscope under **a** control, **b** ATL treatment. Respective time stages of observation are provided at top of the photographs. Arrows indicate apoptotic bodies

Effect on morphology of cells after the lectin treatment could clearly be seen on microscopic examination of cells. A comparison of treated cells with control cells indicated time dependent effect (Fig. 7). After 24 h of treatment, cells started to lose their normal shape and a negative impact on cell number could also be observed. At 48 h, cells became rounded and later after prolonged treatment (72 h) apoptotic bodies as a result of blebbing could be observed in case of ATL treatment. Negative impact of treatment on cell number was clearly visible as was also suggested in trypan blue exclusion assay and DNA damage assay.

## Discussion

The present study was designed to explore the carbohydrate binding behavior and anti-proliferative potential of ATL. In the previous study, using hapten inhibition assay, an interesting carbohydrate binding behavior was observed for ATL. Unlike other araceous lectins, it did not show any binding specificity to mannose, but to a complex glycoprotein Afet. In the present study, the sigmoidal curve obtained in ITC highlighted a cooperative and saturable binding pattern of ATL and Afet. High negative free energy accompanied with the binding indicated a spontaneous binding. This further suggested involvement of hydrophobic forces like hydrogen bonds and van der Waals interactions in ATL–Afet association. Multivalency of Afet dictates the value of *n*, which represents its functional and not structural valency towards ATL. From an *n* value of 0.32 (close to 0.33), a probable trivalent (0.32 = 1/3) cross linked lectin–ligand complex is assumed to be formed between ATL and Afet with a binding ratio of 1:3, indicating that Afet is functionally trivalent for ATL. Low *n* values are inversely correlated to higher binding affinity [20] and a few lectins have been known to show this binding pattern where *n* is less than one for multivalent ligands [21, 22]. The characteristic thermodynamic pattern of ATL–Afet binding can be attributed to one or both factors of ATL having multiple and diverse binding sites (discussed in details through further segments).

The glycan array analysis confirmed upon the previous hapten inhibition assay and present ITC findings with ATL showing highest affinity towards complex glycans. The binding of ATL to various groups was observed to be facilitated by attachment to the core pentasaccharide at various antennary positions. The most common motif amongst all the binding glycans was trimannosyl core pentasaccharide or Manα1-3(Manα1-6)Manβ1-4GlcNAcβ1-4GlcNAcβ (glycan no. 51). The binding affinity was influenced by specific groups attached or even the type of spacer arm (glycan no. 54, 365, 370, 388). Although ATL phylogenetically belongs to the group of lectins which have mannose specificity, but, in corroboration of the previous hapten inhibition assay, ATL did not show any specificity towards mannose (glycan no. 14) alone or even branched mannose (glycan no. 214, 215, 314, 315, 316). Various other interesting features were also observed in glycan array data analysis. Mannose at antennary positions showed affinity only when attached through core pentasaccharide (glycan no. 50, 211, 212, 216, 217, 485). Of these high mannans, 217 and 216 were documented as part of gp120 of HIV-I and glycoprotein antigen CA-125 of epithelial ovarian cancer cells respectively [23, 24]. High mannan glycans have been found to be elevated in both invasive and non-invasive type breast cancer cells when compared to the normal breast epithelial cells [25]. In addition, alterations in high mannose *N*-glycans remain a notable feature of ovarian and liver cancers [2]. In another study, Jacob et al. [26] reported specific binding of anti-glycan antibodies from ovarian cancer patients to high mannose structures printed on glycan array. High specificity of ATL towards high mannose *N*-glycans validates its anti-proliferative potential due to specific glycan binding.

Besides high mannose *N*-glycans, other type of glycans showing high affinity towards ATL were complex *N*-glycans. These constituted glycans having other sugar residues attached at antennary positions. A few of such glycans showed very strong affinity towards ATL (glycan no. 289, 325, 365, 388, 346, 404, 446). ATL has potential insecticidal properties [17]. Glycan array finding indicates that this property may be due to its binding capacity to specific insect surface antigens. The glycan no. 50 to which ATL shows 67% RFU, also known as paucimannose, is commonly found in insects and nematodes [27] whereas glycan 485 is frequently a part of insect glycoproteins [28]. All types of human influenza A and B viruses have been reported to bind to mammalian cells with terminal sequence of Neu5Acα2-6Galβ1-4 [29]. The glycan 346 to which ATL shows 39% RFU, has this terminal sequence attached to core pentasachharide warranting further exploration of this potent molecule to investigate its therapeutic potential against influenza virus.

In addition to these, ATL also showed specificity towards fucosylated core *N*-glycans, although with lower binding affinity (glycans no. 354, 355, 473, 575, 576). Fucosyltransferase enzyme gene responsible for core fucosylation of *N*-glycans is upregulated in various carcinomas [30]. Expression of fucosylated *N*-glycans has been reported to be associated with the events of transformation in hepatoma and cancers related to digestive tract [10, 31]. This has encouraged the application of fucosylated *N*-glycans as markers for respective cancers. ATL was observed to bind with glycan no. 473, which has Lewis X antigen (Fucα1-2Galβ1-4(Fucα1-3)GlcNAcβ-) at the terminal position of a fucosylated *N*-glycan and is being employed as a bladder cancer marker [32]. Lewis antigens and their aberrant expression have been viewed as one of the underlying mechanisms for metastasis in different carcinomas and are considered tumor-associated markers [33]. In addition, lactosamine unit (Galβ1-4GlcNAc) of glycoconjugates is synthesized by β1,4-galactosyltransferase [34]. This enzyme has been found to be upregulated in various cancers and malignant transformations [3, 35]. ATL has shown specificity towards many glycans having terminal lactosamine unit attached to core pentasaccharide at antennary positions forming complex *N*-glycans (glycan no. 54, 352, 354, 355, 365, 370, 388, 398, 576). Interestingly, ATL binds relatively strongly to lactosamine when present only at terminal antennary positions and shows insignificant binding when internally located (glycan no. 271, 309, 321, 372, 428, 483, 484, 577, 608 etc.) or in the form of polylactosamine units (glycan no. 542, 545, 546, 548, 578, 585, 604, 608 etc.).

Bioinformatics analysis indicated that ATL is synthesised as a secretory protein with a N-terminal signal peptide. Mature ATL polypeptide undergoes another C-terminal cleavage and has two domains each having a conserved CRS. Considering these findings and from an earlier SDS-PAGE study of ATL [17], it can be understood that the two such polypeptides of ATL with non-identical subunits should have together oligomerized and resulted in a heterotetrameric protein. Thus ATL presents a configuration of 2 × (A + B) instead of 2 × (A + A) as of the representative lectin of the family—GNA. This heterotetrameric configuration is acquired through tandem gene duplication and sequence divergence over the course of evolution [36, 37]. Sharma et al. [38] argued that the BP2 fold lectins, in this case, ATL, compensated for the loss of two functional units (CRS) in comparison to three of GNA retaining only one, by acquiring multiple subunits. This argument was based on the observation that most of the BP2 lectins have anti-insect potential and they are benefitted by multivalency that they can exhibit accounted for by the multiple subunits [39].

The sequence alignment of ATL with some mannose binding and non-mannose binding Araceae lectins highlights a variation in the sequence of latter ones around CRS. Alanine present downstream of the CRS in the mannose binding lectins is substituted by glutamine, a bulky and charged amino acid residue. Shetty et al. [37] emphasized upon the critical significance of this position in order to accommodate mannose in the CRS and allowing the formation of appropriate hydrophobic contacts with α-d-mannose. It is evident that any substitution at this position will render the lectin incapable of binding to mannose. This revelation at molecular level confirms the findings of sugar inhibition [17] and glycan array analysis of ATL sugar specificity. This is also in consonance with the findings for the other Araceae lectins such as *Sauromatum guttatum*, RVL, *Arum maculatum* agglutinin and *Lysichiton camtschatcensis* lectin [21, 34, 37, 40] which, similar to ATL do not show specificity towards simple mannose. The homology modeling besides CDD and Prosite also predicted ATL to have two GNA-like domains with single CRS per domain having conserved BP2 architecture. A good quality model was indicated by acceptable error functions and a good number of residues with-in the verified 3D environment. The superior quality of homology model was supplemented by a high Z-score and an impressive Ramachandran plot. The residues in generously allowed and disallowed regions were only located in the loops relieving the concerns regarding their interference in the functioning of the proposed homology model. The G-factors for ATL model were also within the usual range reaffirming the homology model quality.

An analysis of evolutionary relationship of 49 taxa across the various orders that were included in the present study, confirmed upon that the GNA-related lectins are not a monophylogenetic group [36, 37]. The lectins clustering under Alismatales branch, including ATL were synthesized as two domain single polypeptide lectins showing sugar specificity to complex carbohydrates rather than simple carbohydrates. Also, these lectins showed common features of anti-proliferative and anti-insect properties [7, 12–15, 17]. A closer examination of the phylogenetic analysis also suggests that all the lectins belonging to order Alismatales including ATL followed a distinct evolutionary course relative to rest of the GNA related lectins and that this event is relatively recent in evolutionary history.

Overall, glycan array and sequence analysis have clearly demonstrated the uniqueness in the binding specificity of ATL. Diversity in the glycan receptors on cell surface of different cells accounts for the variations in the cytotoxicity/anti-proliferative behavior of different lectins. These variations can explain the differential binding of ATL to different cancer cells and hence diversity in its anti-proliferative potential. It is well known that plant lectins mediate their anti-proliferative properties due to their capability to bind to foreign glycans [7, 10]. Thus a lack of receptors for ATL on the surface of A498 cells could explain the non-responsiveness of the latter towards the treatment of former. A similar explanation is justifiable for the varied effect of ATL on HeLa and A549 cells where ATL was non-responsive towards HeLa and inhibited only 21% proliferation of the A549 cells. Similarly, a variation in the extent of proliferation inhibition was observed in case of HCT-15, SW-620 and COLO-205 (colon) and A549^3^ and HEP-2 (lung) cancer cell lines towards the treatment of ATL in previous study [17]. This finding further strengthens the fact that same lectin has different effects on different cancer cells.

Expected pattern in decrease of nucleic acid content was observed which was parallel to the results of trypan blue assay and DNA damage assay. Pronounced DNA damage, one of the well studied hallmarks of the apoptotic cells, was observed at 72 h of treatment. However, the DNA damage induced by ATL was not observed in the form of classic DNA laddering assay. This absence of oligonucleosomal DNA ladder is a characteristic feature of IMR-32 cells undergoing apoptotic death [41]. As mentioned by Yuste et al. [42], IMR-32 cells find themselves in the category of other cells including IMR-5 (neuroblastoma), NT2 (human neuronal like cells), DU145 (prostate cancer) and MCF-7 (breast cancer), that never show typical DNA ladder that appears as a result of caspase-activated DNases during apoptotic cell death. In case of IMR-5 cells, it was reported that caspase activated DNases are responsible for high molecular weight nucleosomal degradation whereas, caspase-3 mediated oligonucleosomal DNA ladder is absent [42]. This literature supports the observation of DNA damage assay in present work and further affirms that ATL treated cells show apoptotic cell death, induced by caspase activated nucleosomal degradation. Morphology assay indicated rounding, chromatin condensation and membrane blebs, characteristic attributes of apoptosis, in the ATL treated cells.

## Conclusion

A complex fine sugar specificity of ATL is reported here. It binds to a range of glycans that have been reported to be expressed on the surface of cancer cells or during malignant transformation, as insect glycoprotein, on the surface of human influenza virus and HIV. ITC demonstrated a spontaneous and cooperative binding of ATL towards its multivalent glycoconjugate ligand. Sequence analysis of ATL also demonstrated that ATL exhibits mutations with-in and downstream of the conserved carbohydrate recognition sites, thus directing its specificity towards foreign glycans usually expressed in viruses, insects or humans. This unique and diverse sugar specificity complemented with multivalency was suggested to be acquired through domain duplication and tandem mutation events. These observations explain different biological properties of ATL such as anti-proliferative, anti-insect and anti-viral potential, which can be exploited for various applications. In addition, immediate work demonstrated that ATL induces apoptosis in the human cancer cells to mediate its anti-proliferative effect. Various investigations establish that this property of the lectin is due to its specific binding property to the aberrant glycans expressed on the surface of cancer cells. Thus, ATL establishes its significance and calls for more robust research on its possible use as anti-proliferative, anti-insect or anti-viral therapeutic candidate.

## Methodology

### Plant material and lectin purification

*Arisaema tortuosum* *var. helleborifolium* Schott belongs to family Araceae and is a seasonal monocot plant growing under wild conditions. It grows in Himachal Pradesh, India to Central Nepal and South East Tibet at an altitude of 1000–2300 m in stony slopes. Sampling was carried out after getting the due consent from Department of Biosciences, Himachal Pradesh University, Shimla. Tubers were collected, stored and processed as described previously [21]. The lectin ATL was purified from crude extract prepared from plant tubers by affinity chromatography [15]. PBS dialysed sample was used for all the experiments.

### ATL lectin–carbohydrate interaction

#### Isothermal titration microcalorimetry

Calorimetric measurements to study the interaction of ATL with Afet were performed with MicroCal™ iTC_200_ (GE Healthcare Life Sciences) as described previously [21]. Enthalpy change (Δ*H*), entropy change (Δ*S*), association constant (*K*_a_) and stoichiometry/no. of ligand binding sites (*n*) were obtained by fitting data into one site model. Following equation was used to perform further calculations of thermodynamic parameters:

$$\Delta G = \Delta H - T\Delta S = - RTlnK_{a}$$

where Δ*G* is the change in free energy, *T* is the temperature and *R* is gas constant having value 1.987 cal mol^−1^ degree^−1^.

#### Glycan array analysis

Facility at Core H, Consortium for Functional Glycomics, USA was availed to perform glycan array analysis (array version 5.0) of biotinylated ATL using the protocol given by Blixt et al. [43]. Experimental details related to the array analysis are same as described previously [21]. The array data was analyzed at Glycopattern (results based on z-score analysis) [44] (https://glycopattern.emory.edu) and by applying ranking analysis [45]. This helped defining a specific binding motif for ATL by assigning a rank to each glycan based upon normalizing the RFUs for each concentration. These ranks were further averaged to obtain highest and lowest binders.

#### Molecular cloning

RACE-PCR was performed on the total RNA isolated from the snap-frozen tubers as described in our earlier work [21]. Primers, PCR cycle and cloning strategies were also same for 3′RACE-PCR. The 3′ nested primers for 5′RLM-RACE PCR designed from the 3′RCAE product were AT_OUTER (5′-CCCTTGTGGTTGAGCCTGAGGAAGC-3′) and AT_INNER (5′-CCACCGTACAGGACCAGGTTGCAG-3′) and 5′ primers were from the kit (FirstChoice^®^ RLM-RACE Kit, Ambion, Life Technologies, USA). A full length construct prepared by joining 3′ and 5′ RACE products was used to further design the forward (AT_FP, 5′-AGCGCCATCACGGCGGAGTAGAAGA-3′) and reverse (AT_RP, 5′-TAACGCAACTCAACAGGTAGCC-3′) primers for the full length PCR. A full length PCR cycle was set under conditions mentioned previously with 67 °C as annealing temperature.

#### Bioinformatics analysis

The sequence obtained after the cloning was subject to various bioinformatics analysis similar to as described in our previous work [21]. MEGA X [46] using Neighbor-Joining method [47] was used to prepare a phylogenetic tree. The evolutionary distances in the tree were computed using the number of differences method [48] and are presented in the units of the number of amino acid differences per sequence. Taxonomical division of the lectins included in the study based on APG III is provided in Additional file 6: Table S4.

Homology models for ATL sequence were constructed using MODELLER 9v15 suite [49] based upon the coordinates of the PDB structures of highest sequence identity published as on 9-3-2016 at SWISS-MODEL workspace (http://swissmodel.expasy.org/). Similar methods of evaluation for the homology models were employed as in previous work [21]. A total of 15 models were analyzed and 3D^refine^ web server was further employed to refine the 3D structure of proposed models [50]. PyMol (www.pymol.org, Version 1.7.4) was used to visualize the protein structure in 3D cartoon.

### Anti-proliferative activity

HeLa (cervical), A498 (kidney), A549 (lung) and IMR-32 (neuroblastoma) cell lines were procured from National Centre for Cell Science, Pune, India. The cultures were maintained at standard conditions at 37 °C in CO_2_ (5%) incubator (Hereaus^®^ BB15, Thermo Scientific) and 3-[4,5-dimethylthiazol-2-yl]-2,5-diphenyl tetrazolium bromide (MTT) assay was set up in the growth medium supplemented with gentamycin, penicillin and streptomycin. For storage of cells, DMSO was added at a concentration of 10% v/v and stored at − 80 °C. For all the further experiments, the lectin concentration used was 100 µg ml^−1^ and observations were made at 0, 24, 48 and 72 h of treatment. All the observations were compared to control set up in parallel and the experiments were set up in triplicates. Cell viability as an effect of lectin treatment in a time dependant manner was assessed by trypan blue assay with cells seeded at a concentration of 1 × 10^4^ cells ml^−1^ in 96 well plates (100 µl/well). At regular intervals mentioned above, cells were removed by trypsinization and counted on hemocytometer.

As an antiproliferative agent, the cell death caused by lectin may result due to underlying DNA damage. To assess this, 2 ml cell suspension at a concentration of 2×10^5^ cells ml^−1^ was added in each well of 6-well plates (Microtest™, Falcon, USA). There were two replicates for each stage. Upon harvesting at prescribed intervals, DNA isolation was performed by organic method [51]. The isolated DNA was electrophoresed (Midi Submarine Electrophoresis Unit, Tarsons, India) in 1% agarose gel with 1× Tris Acetic acid EDTA running buffer at 50 V. DNA was viewed with ethidium bromide under UV gel documentation system (AlphaImager Mini, Cell Biosciences, USA).

In order to study the probable changes in morphology of the lectin treated cells, 1 ml cell suspension was seeded at a concentration of 1 × 10^5^ cells ml^−1^ in each well of the 12-well plate having a lysine coated coverslip. Fetal bovine serum supplemented medium replaced lectin in control wells. After each treatment period, medium was aspirated from respective wells. Cells were washed with 1 ml ice cold PBS, followed by two washings each of 1 ml PBS at room temperature. Cells were fixed by incubating in paraformaldehyde for 20 min and removed by rinsing the cells twice with PBS. Coverslips were inverted on previously mounted flouromount™ (Sigma, Aldrich) on a glass slide. Fixed cells were viewed and photographed under the phase contrast microscope (Nikon AIR Confocal LSM, Japan).

## Additional files

**Additional file 1: Table S1.** Thermodynamic parameters for the interaction of ATL with asialofetuin.

**Additional file 2: Figure S1.** Full length coding sequence of lectin genes from mRNA of ATL. Numbers indicate ORF. Deduced amino acid sequence in one letter code are mentioned below the coding sequence. The start and stop codons are highlighted in bold and shade. The upright arrow indicates predicted cleavage site for signal peptide. Italicised amino acid sequence represents conserved bulb-type lectin DOM 1 and 2. Adjacent cysteins predicted to be involved in disulphide linkage in respective domains are highlighted in shadow. Conserved CRS [QXDXNXVXY] are indicated in bold and are boxed.

**Additional file 3: Table S2.** Predicted physico-chemical properties of ATL.

**Additional file 4: Table S3.** Accession numbers of lectins and their full forms included in this comparison.

**Additional file 5: Figure S2.** (**A**) Cartoon representation of theoretical model of predicted ATL polypeptide as visualized by PyMol. Sheets are represented as red ribbons and loops as magenta lines. The conserved CRS are in green sticks. (**B**) Overall model quality and **(C)** Local model quality graphs for ATL model as determined by ProSAII. (**D**) Ramachandran plot of final ATL model as determined by PROCHECK. Blue squares represent non-glycine residues, blue triangles represent glycine residues and red squares represent residues that are not favoured in Ramachandran plot.

**Additional file 6: Table S4.** Taxonomical division of lectins included in phylogenetic analysis.

## Data Availability

The original glycan array data for ATL is available at the Consortium for Functional Glycomics website (http://www.functionalglycomics.org/) with the ID cfg_rRequest_2313. The coding sequence of ATL mRNA and deduced protein sequence is available with GenBank accession number KX132810 and APQ47296 respectively. All other data generated or analyzed during this work, significant in order to understand the work, are included in this published article (and its Additional files).

## Abbreviations

Afet: asialofetuin
ATL: *Arisaema tortuosum* lectin
BP2 lectin: beta prism 2 lectin
CEA: *Colocasia esculenta* agglutinin
CRD: carbohydrate recognition domain
CRS: carbohydrate recognition site
GNA: *Galanthus nivalis* agglutinin
ITC: isothermal titration microcalorimetry
RACE: rapid amplification of cDNA ends
RFU: relative fluorescence unit
RLM-RACE: RNA ligation mediated rapid amplification of cDNA ends
RVL: *Remusatia vivipara* lectin

## Footnotes

1 * Significant at p < 0.01.

2 ** Significant at p = 0.000.

3 Tested in present work with MTT assay.
