# Supplementary material for: Exploration of carbohydrate binding behavior and anti-proliferative activities of Arisaema tortuosum lectin
Source: BMC Mol Biol. 2019 May 7;20:15. doi: 10.1186/s12867-019-0132-0 (PMC6505227; doi:10.1186/s12867-019-0132-0)
Supplement: Supplementary file 1 — Additional file 1: Table S1. Thermodynamic parameters for the interaction of ATL with asialofetuin. [file 12867_2019_132_MOESM1_ESM.docx]

**Additional file 1: Table S1**

Thermodynamic parameters for the interaction of ATL with asialofetuin

| **Thermodynamic parameters*** | **ATL** | |
| --- | --- | --- |
| **Δ*H***  (kcal mol^-1^) | | -40.79 |
| **Δ*S***  (kcal mol^-1^deg^-1^) | | -0.114 |
| ***T*Δ*S***  (kcal mol^-1^) | | -2.85 |
| **Δ*G***  (kcal mol^-1^) | | -37.94 |
| ***K*_a_**  (M^-1^) | | 1.16 × 10^5^ |
| ***n***  (binding sites per monomer) | | 0.32 |

*Δ*H* = Enthalpy change, Δ*S* = Entropy change, Δ*G* = Free energy change,

*K*_a_ = Association constant
