# Supplementary material for: Exploration of carbohydrate binding behavior and anti-proliferative activities of Arisaema tortuosum lectin
Source: BMC Mol Biol. 2019 May 7;20:15. doi: 10.1186/s12867-019-0132-0 (PMC6505227; doi:10.1186/s12867-019-0132-0)
Supplement: Supplementary file 2 — Additional file 2: Figure S1. Full length coding sequence of lectin genes from mRNA of ATL. Numbers indicate ORF. Deduced amino acid sequence in one letter code are mentioned below the coding sequence. The start and stop codons are highlighted in bold and shade. The upright arrow indicates predicted cleavage site for signal peptide. Italicised amino acid sequence represents conserved bulb-type lectin DOM 1 and 2. Adjacent cysteins predicted to be involved in disulphide linkage in respective domains are highlighted in shadow. Conserved CRS [QXDXNXVXY] are indicated in bold and are boxed. [file 12867_2019_132_MOESM2_ESM.docx]

**Additional file 2: Figure S1**

75 **atg**gccaaactcctcctcttcctcctcccggccatcctcggcctc

**M** A K L L L F L L P A I L G L

120 gtcattcctcgctcagccgcggcagtgggaaccaactacctgctg

V I P R S A A A V G T *N Y L L*

165 tccggcgaaaccctaaacaagaacgaccatctcaggaacggcgac

*S G E T L N K N D H L R N G D*

210 ttcgacctggtcatgcaggaagactgcaacgccgtcatgtacaat

*F D L V M* ***Q*** *E* ***D*** *C* ***N*** *A* ***V*** *M* ***Y*** *N*

255 ggcaattggcaatccaacacggccaacaagggacgagactgcaag

*G N W Q S N T A N K G R D C K*

300 ctcactctgaccgaccgcggcgagctcctcatcaaaaatggcgcc

*L T L T D R G E L L I K N G A*

345 ggatccacagtctttagcagcggctcccagtccgtcaggggcaac

*G S T V F S S G S Q S V R G N*

390 tacgctctcgtcgtccgtccggccgatgggagactggtcatctac

*Y A L V V R P A D G R L V I Y*

435 ggcccgtccgtcttcgagattaacccttgggtccccggcctgaac

*G P S V F E I N P W V P* G L N

480 agcctgcggcaccccgacatcccgatcacgaacaacatgctcttc

S L R H P D I P I T N *N M L F*

525 tccggccaagtcctgtacggcgacggcatgctcgttgcgaggaac

*S G Q V L Y G D G M L V A R N*

570 cacaggctcgtcatgcagggcgactgcaacctggtcctatacggt

*H R L V M* ***Q*** *G* ***D*** *C* ***N*** *L* ***V*** *L* ***Y*** *G*

615 ggcaaattcggttggcagtccaacacccacggcaacggcgagcac

*G K F G W Q S N T H G N G E H*

660 tgcttcgtcaggttgaaccacaagggcgagctcgtcttcaaggac

*C F V R L N H K G E L V F K D*

705 gacgacttccagaccatctggagcagccgatccagctccaccaag

*D D F Q T I W S S R S S S T K*

750 cagggtgactacgccttcatcctccaggacaacggcttcggcgtc

*Q G D Y A F I L Q D N G F G V*

795 atctacggccctgccatctgggagactagctcgaagcgcgccatt

*I Y G P A I W E T S S K R A* I

840 gcggcg**tag** 848

A A *****

**Fig. 3** Full length coding sequence of lectin genes from mRNA of ATL. Numbers indicate ORF. Deduced amino acid sequence in one letter code are mentioned below the coding sequence. The start and stop codons are highlighted in bold and shade. The upright arrow indicates predicted cleavage site for signal peptide. Italicised amino acid sequence represents conserved bulb-type lectin DOM 1 and 2. Adjacent cysteins predicted to be involved in disulphide linkage in respective domains are highlighted in shadow. Conserved CRS [QXDXNXVXY] are indicated in bold and are boxed.
