# Supplementary material for: Exploration of carbohydrate binding behavior and anti-proliferative activities of Arisaema tortuosum lectin
Source: BMC Mol Biol. 2019 May 7;20:15. doi: 10.1186/s12867-019-0132-0 (PMC6505227; doi:10.1186/s12867-019-0132-0)
Supplement: Supplementary file 3 — Additional file 3: Table S2. Predicted physico-chemical properties of ATL. [file 12867_2019_132_MOESM3_ESM.docx]

**Additional File 3: Table S2**

Predicted physico-chemical properties of ATL.

| **Physico-chemical properties** |  |
| --- | --- |
| No of amino acids | 234 |
| Molecular weight (kDa) | 25.8 |
| Theoretical pI | 6.64 |
| No. of negative residues | 22 |
| No. of positive residues | 21 |
| Extinction coefficient* (M^-1^ cm^-1^) | 39670 |
| Estimated half life in *E.coli* | >10 h |
| Instability index | 28.43 |
| Aliphatic index | 79.1 |
| GRAVY score | -0.350 |

*assuming all residues of Cys residues for Cystines
