# Supplementary material for: Exploration of carbohydrate binding behavior and anti-proliferative activities of Arisaema tortuosum lectin
Source: BMC Mol Biol. 2019 May 7;20:15. doi: 10.1186/s12867-019-0132-0 (PMC6505227; doi:10.1186/s12867-019-0132-0)
Supplement: Supplementary file 4 — Additional file 4: Table S3. Accession numbers of lectins and their full forms included in this comparison. [file 12867_2019_132_MOESM4_ESM.docx]

**Additional file 4: Table S3**

**Accession numbers of lectins and their full forms included in comparison**

| **Sr. No.** | **Lectin** | **Abbreviation** | **Accession No.** | **Family** |
| --- | --- | --- | --- | --- |
| 1 | *Colocasia esculenta* | CEA | BAA03722 | Araceae |
| 2 | *Remusatia vivipara* | RVL | ACH41914 | Araceae |
| 3 | *Tulipa* hybrid cultivar | Tulipa | AAC49387 | Liliaceae |
| 4 | *Galanthus nivalis* | GNA | AAL07474 | Amaryllidaceae |
| 5 | *Lysichiton camtschatcensis* | LCL | BAL44282 | Araceae |
| 6 | *Arisaema tortuosum* | ATL | APQ47296 | Araceae |
