# Supplementary material for: Exploration of carbohydrate binding behavior and anti-proliferative activities of Arisaema tortuosum lectin
Source: BMC Mol Biol. 2019 May 7;20:15. doi: 10.1186/s12867-019-0132-0 (PMC6505227; doi:10.1186/s12867-019-0132-0)
Supplement: Supplementary file 5 — Additional file 5: Figure S2. (A) Cartoon representation of theoretical model of predicted ATL polypeptide as visualized by PyMol. Sheets are represented as red ribbons and loops as magenta lines. The conserved CRS are in green sticks. (B) Overall model quality and (C) Local model quality graphs for ATL model as determined by ProSAII. (D) Ramachandran plot of final ATL model as determined by PROCHECK. Blue squares represent non-glycine residues, blue triangles represent glycine residues and red squares represent residues that are not favoured in Ramachandran plot. [file 12867_2019_132_MOESM5_ESM.docx]

**Additional File 5: Figure S2**

**
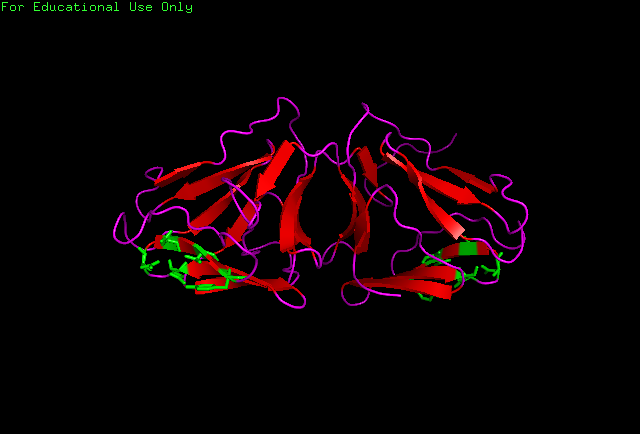
**

**(A)**

**
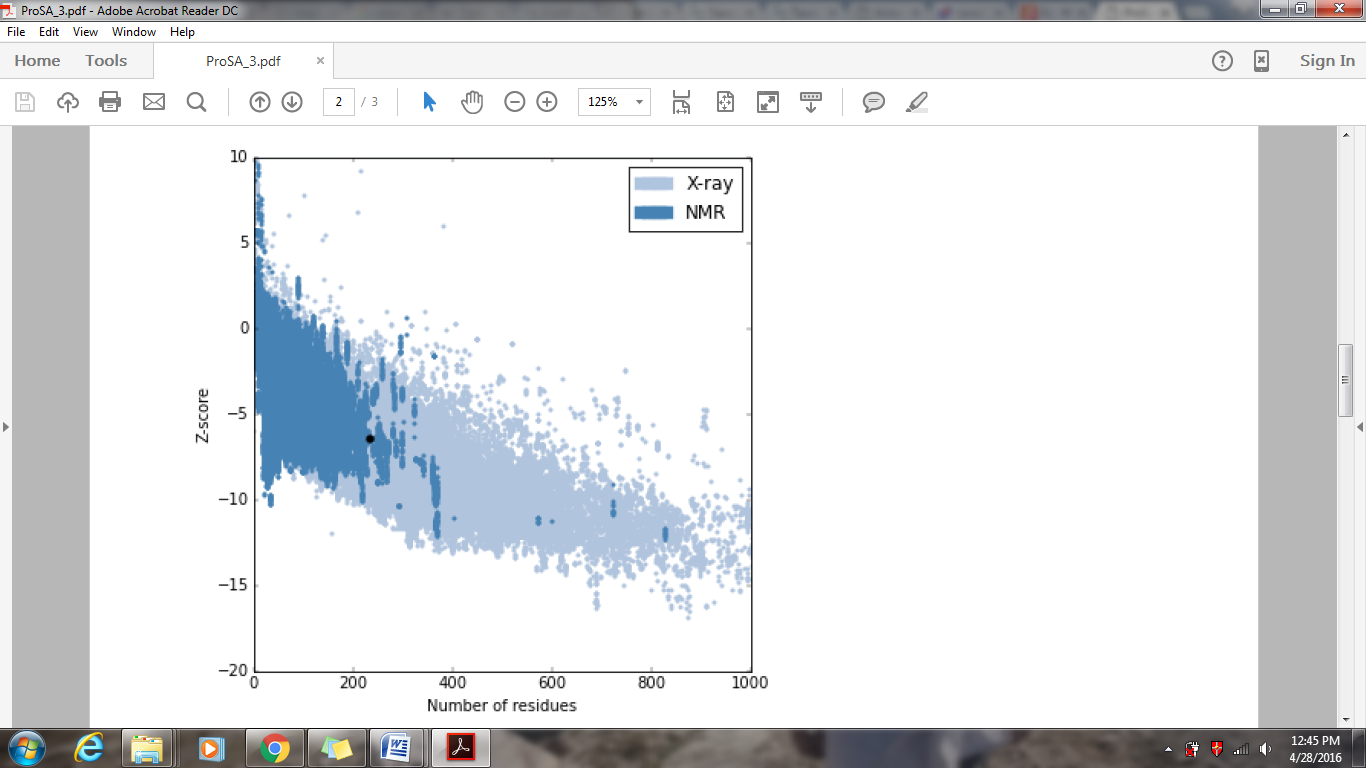

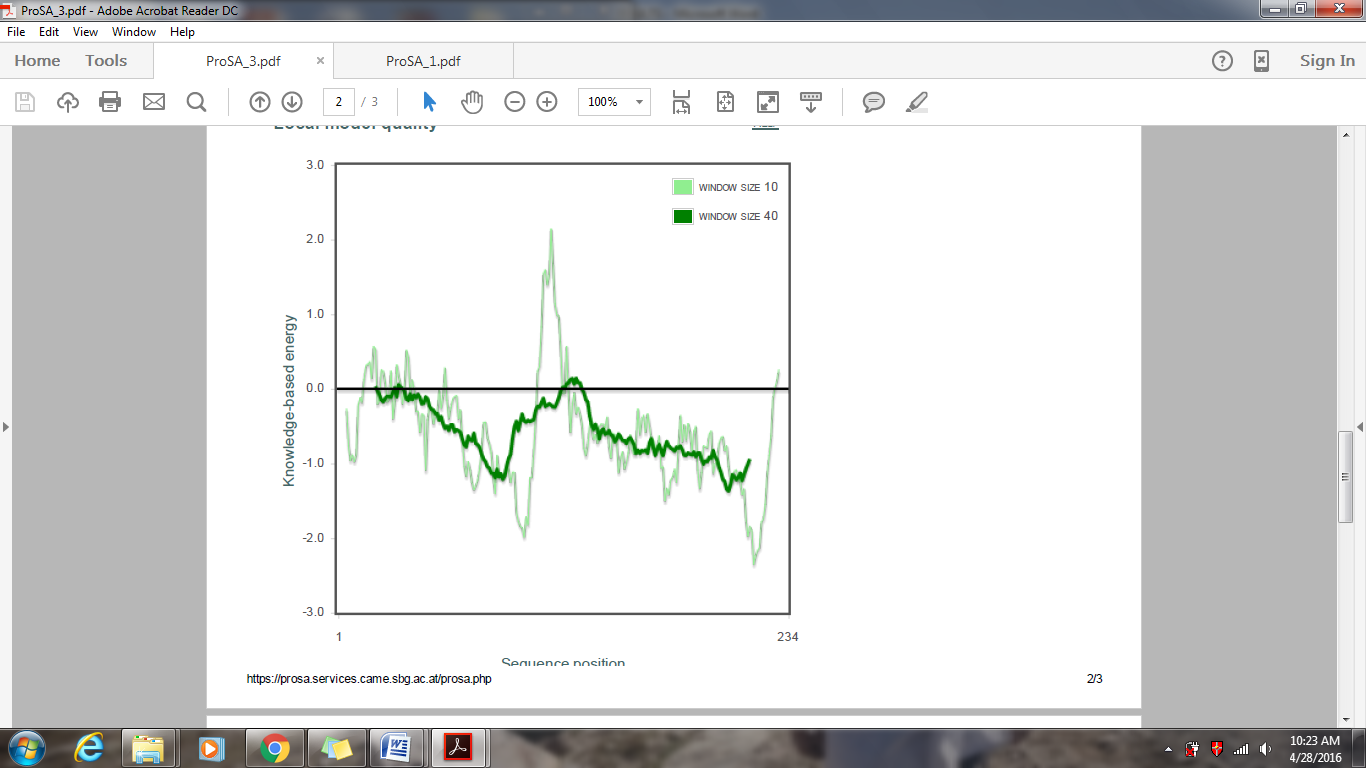
**

**Z-score**

**Knowledge-based energy**

**Sequence position**

**No. of residues**

**(B) (C)**

**
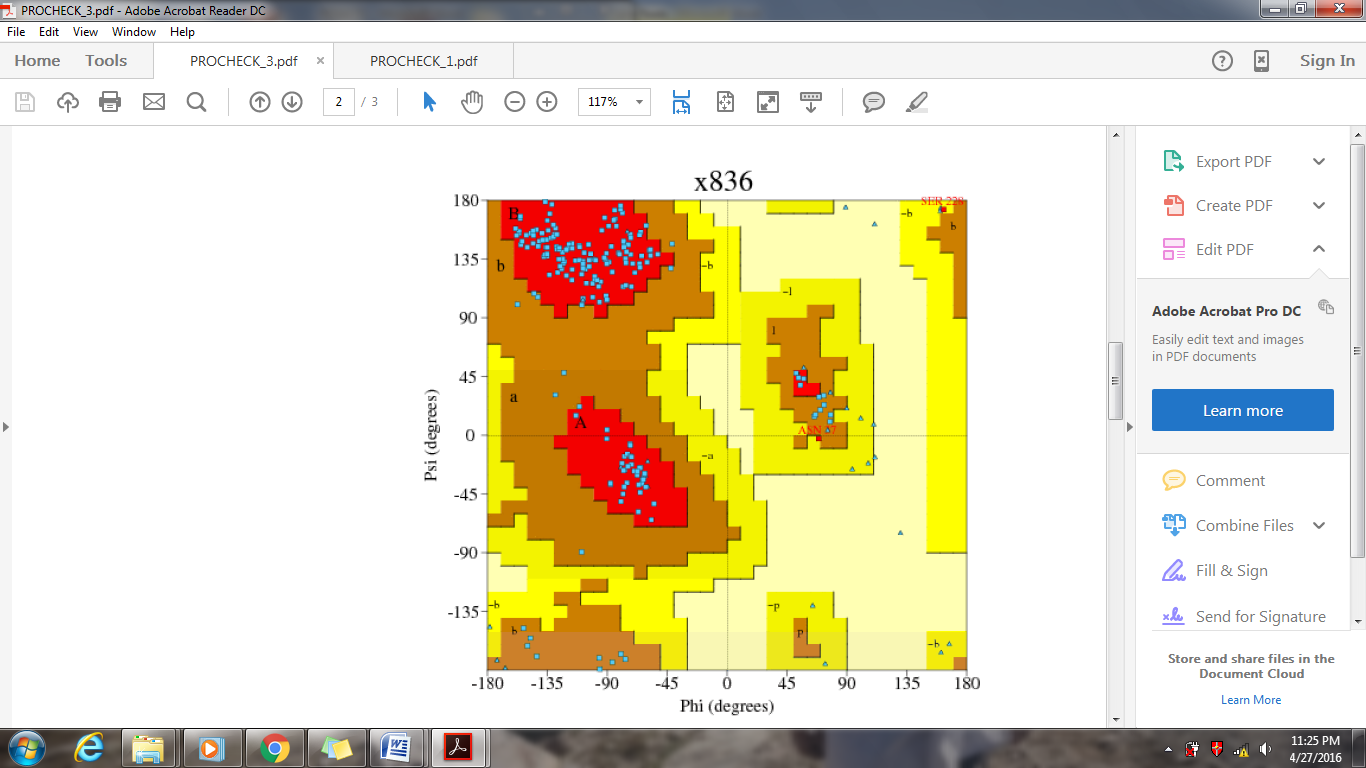
**

**(D)**

**(A)** Cartoon representation of theoretical model of predicted ATL polypeptide as visualized by PyMol. Sheets are represented as red ribbons and loops as magenta lines. The conserved CRS are in green sticks. **(B)** Overall model quality and **(C)** Local model quality graphs for ATL model as determined by ProSAII. **(D)** Ramachandran plot of final ATL model as determined by PROCHECK. Blue squares represent non-glycine residues, blue triangles represent glycine residues and red squares represent residues that are not favoured in Ramachandran plot.
