# Supplementary material for: Exploration of carbohydrate binding behavior and anti-proliferative activities of Arisaema tortuosum lectin
Source: BMC Mol Biol. 2019 May 7;20:15. doi: 10.1186/s12867-019-0132-0 (PMC6505227; doi:10.1186/s12867-019-0132-0)
Supplement: Supplementary file 6 — Additional file 6: Table S4. Taxonomical division of lectins included in phylogenetic analysis. [file 12867_2019_132_MOESM6_ESM.docx]

**Additional file 6: Table S4**

**Taxonomicasl division of lectins included in phylogenetic analysis**

| **Sr. No.** | **Taxonomic division** | **Accession No.** |
| --- | --- | --- |
| 1. | Gymnosperm | AAZ30388, AAT73201 |
| 2. | Angiosperm | |
|  | a. Dicot | AGI04215, ADG04234, AAZ30387, AAZ30386, AAD03580, ACD13798, AGL46982 |
|  | b. Monocot | AAB35217, AAC49387, ADK55603, AAZ30385, AAZ30383, AAZ30382, ACR15122, AAP57409, ADX01383, AAK29077, BAL44282, ABY91323, BAD67184, AAM28644, CAB94238, AAQ75079, AGL46982, COHJM8, AAP20877, AAV70492, AAC48927, AAV66418, AAL07474, AAP22169, AAM28277, AAC48997, BAA03722, AAP50524, ABX47148, ABM68041, AAS66304, ACH41914, ABC69036, BAE45253, ABM74186, AAA32646, AAD16404, ASR80892, APQ47297, APQ47296, AAQ18904 |
